# A Study to Check the Readiness of Final-Year Dental Students Vis-à-Vis to a Safe and an Independent Practitioner in Sultanate of Oman Amidst the Recent Pandemic

**DOI:** 10.1155/2024/7476437

**Published:** 2024-10-15

**Authors:** Triveni Nalawade, Sanjay Saraf, Rachappa Mallikarjuna, Belal Haj-Hamed, Siva Kumar, Nutayla Al Harthy, Mohamed Al Ismaily

**Affiliations:** ^1^Paediatric Dentistry, Child Dental Health, Oman Dental College, Muscat, Oman; ^2^Oral Pathology, Oman Dental College, Muscat, Oman; ^3^Oman Dental College, Muscat, Oman; ^4^E-Learning, Oman Dental College, Muscat, Oman; ^5^Adult Restorative Dentistry, Oman Dental College, Muscat, Oman; ^6^Oral and Maxillofacial Surgery, Oman Dental College, Muscat, Oman

**Keywords:** COVID-19, dental surgical procedures, DU-PAS score, professionalism, self-perceived preparedness

## Abstract

It is essential for a dental student about to graduate to possess robust fundamentals in both basic and clinical dental science. There should be a perfect harmony and blend of basic and clinical science integration and application among final year graduating students. They should also demonstrate readiness and competence in performing various clinical skills independently, after correctly interpreting and diagnosing a dental disorder. Besides the clinical aspects of dentistry, the newly graduating dental student should understand the principles of professionalism and ethos. The main aim and objective were to conduct a study to assess the impact of COVID-19 on practice readiness among the fifth-year dental students at Oman Dental College (ODC). A study was conducted to measure self-preparedness among graduating students, especially during the challenging times of the COVID-19 pandemic when education was significantly impacted. This study used a validated Dental Undergraduates Preparedness Assessment Scale (DU-PAS). The DU-PAS consists of 50 items, including 24 items related to clinical skills (part A) and 26 items related to scientific knowledge and affective skills (part B). Each item in part A was scored on a three-point scale ranging from no experience (0), with verbal and/or practical input from a colleague (1), to independently (2). Items in part B of the DU-PAS are scored on a three-point scale: no experience (0), mostly (1), and always (2). The maximum score for the 50 items on the DU-PAS is 100. Interestingly, the current study showed that the ODC final-year students were confident in performing several clinical procedures and had a good understanding of various professional aspects of clinical dentistry. The didactic online classes and preclinical sessions conducted while adhering to social distancing and other safety norms during the COVID-19 pandemic, were positively received by the students.

## 1. Advances in Knowledge

Due to the COVID-19 pandemic, many changes occurred in the teaching and learning of these COVID cohorts. The first cohort, which graduated in 2020–21, needed to be assessed to ensure they were safe beginners and independent practitioners. This study helped ensure that the students, who would become part of the Sultanate of Oman's dental workforce were clinically prepared and well-equipped to face the challenges in clinical dentistry.

## 2. Application in Patient Care/Student Learning-Teaching

The shortcomings and other deficiencies noticed during the assessment can be incorporated into the curriculum review, and attempts can be made to address these issues for the next academic year's (AY's) students. The current study showed less preparedness among graduating students in performing procedures like amalgam cavity preparation and restoration. To counteract these deficiencies, a training workshop on amalgam restoration, its repair and replacement, and precautions during amalgam disposal was conducted for the current graduating batch during their summer clinics. Due to these changes in the dental graduates' curriculum, future dentists will be much better prepared for independent practice, thereby improving patient care. As the pandemic is over and normalcy has returned, the study's immediate relevance may be minimal. However, in a similar situation in the future, the results could help minimize adverse effects.

## 3. Introduction

Globally, the COVID-19 pandemic impacted all aspects of life, including social, economic, and psychological spheres. The shift from face-to-face teaching to online and distant learning significantly affected teaching, and consequently, student learning. Oman Dental College (ODC) is the sole institution in the Sultanate of Oman where dental science education is imparted over 5 years. The course is divided annually, with year I preceded by the zero or foundation year. This preparatory foundation year begins with the recapitulation and reinforcement of applied English language, information technology, and mathematics. Formal dental teaching starts with the study of basic medical sciences, followed by an introduction to basic dental and preclinical dental sciences. Clinical dental science subjects are introduced in year III, followed by elaborate didactic, practical, and clinical teaching, continuing until the course culminates in year V. Throughout the course, ODC students are also instilled with ethical values, professionalism, and team-working skills. By the end of their program, final-year students are competent in their professional skills, communication, clinical management, and leadership, in conformity with the expectations of the General Dental Council (GDC) and the Association for Dental Education in Europe [1, 2]. The aim of the undergraduate course is to prepare final-year students to practice independently in a safe and effective manner [3, 4].

An ideal dental school curriculum should include teaching students how to solve real-life situations, build ethical relationships with patients, and instill practice-based learning [5]. On the other hand, a dental undergraduate student needs to be confident, ready, and prepared to transition smoothly from a dental institution to the field of dental practice. Effective pedagogy, curriculum design, and teaching methods are the best routes to ensure graduates' preparedness for their future careers [6, 7]. It is also equally important to assess the work preparedness perceived by an undergraduate final-year dental student [5].

At the close of 2019, a menacing pneumonia caused by a novel virus (SARS-CoV-2) emerged, spanning almost all habitable countries and posing a threat to all of mankind. Oman reported its first COVID-19 cases on February 24, 2020, among travelers who had returned home from Iran [8, 9]. The COVID-19 disease stalled all aspects of life globally, including education. To limit the spread of the disease, the Ministry of Education in Oman closed all higher education colleges and schools on March 15, 2020, and encouraged online virtual or hybrid modes of teaching, including ODC, a stand-alone dental institute [10]. This lockdown affected the teaching of onsite basic medical/dental sciences, preclinical dental sciences, and applied clinical dental sciences. Traditional face-to-face lectures, preclinical, and clinical sessions were temporarily replaced by distance learning using an online platform, like other dental schools worldwide [5].

This unusual event did not affect the final-year 2019–2020 batch, as they were largely in the preparatory stage of their final summative exams. However, the next final-year 2020–2021 batch, which was supposed to start in September 2020, bore the maximum brunt of the pandemic closure. The new AY 2020–2021 for the final-year students started in September 2020 with only online lectures at the outset, followed by hybridized online and onsite preclinical and clinical teaching after a few months. This group of students attended the dental institute in batches or groups on alternate days, observed social distancing, and donned masks while working on simulators and models. However, this special batch did work on patients with all safety precautions and avoided treatments that generated aerosol.

There are numerous studies assessing the self-perceived preparedness of undergraduate dental students, including those conducted during the COVID-19 pandemic, which impacted the education sector. The main aim and objective were to assess the impact of COVID-19 on the practice readiness of fifth-year dental students at ODC. The change in teaching and learning methods, such as online classes and distant learning, affected student learning, but whether it impacted the practice readiness of graduating dental students was the primary reason for conducting this study.

## 4. Methods

Ethical approval was obtained from the Ethical Research Committee of ODC (ODC Research 2021_5) prior to the conduct of the study. The study was conducted in accordance with the guidelines of the Declaration of Helsinki. Purposive sampling was used to recruit the final-year dental undergraduate students at ODC, who were invited to participate in an online questionnaire-based study using a validated Dental Undergraduates Preparedness Assessment Scale (DU-PAS) [3]. The DU-PAS is a validated questionnaire to measure the various clinical and soft skills expected from graduating dental students. The DU-PAS is a validated questionnaire designed to measure the various clinical and soft skills expected from graduating dental students. It consists of part A and part B, with a total score ranging from 0 to 100 [1].

Part A of the scale consists of 24 questions related to clinical skills and is scored on a 3-point scale ranging from zero to two, as summarized in Table 1, as follows:

Part B consists of 26 questions related to soft skills and is scored on a 3-point scale ranging from zero to two, as summarized in Table 2, as follows:

The questionnaire created using Microsoft forms was sent via a link through official email to all final-year undergraduates' institutional email IDs, along with an information sheet detailing the study's methodology, benefits and risks, confidentiality, voluntary participation, ethical approval, and contact information for the principal investigators. Only final-year students were included in the study, and incomplete forms were excluded. The study was conducted using an online questionnaire, preceded by a participant information sheet summarizing its aims. Students who attempted the questionnaire were considered to have given their consent. Participants were given 1 week to respond to the questionnaire. A reminder was sent twice, at 2-day intervals, after they received the initial online questionnaire. No further reminders were sent.

## 5. Results

**Figure 1:**
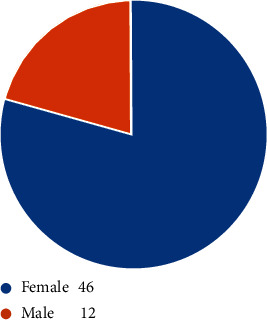
Pie chart showing distribution by gender amongst the final-year dental students.

**Figure 2:**
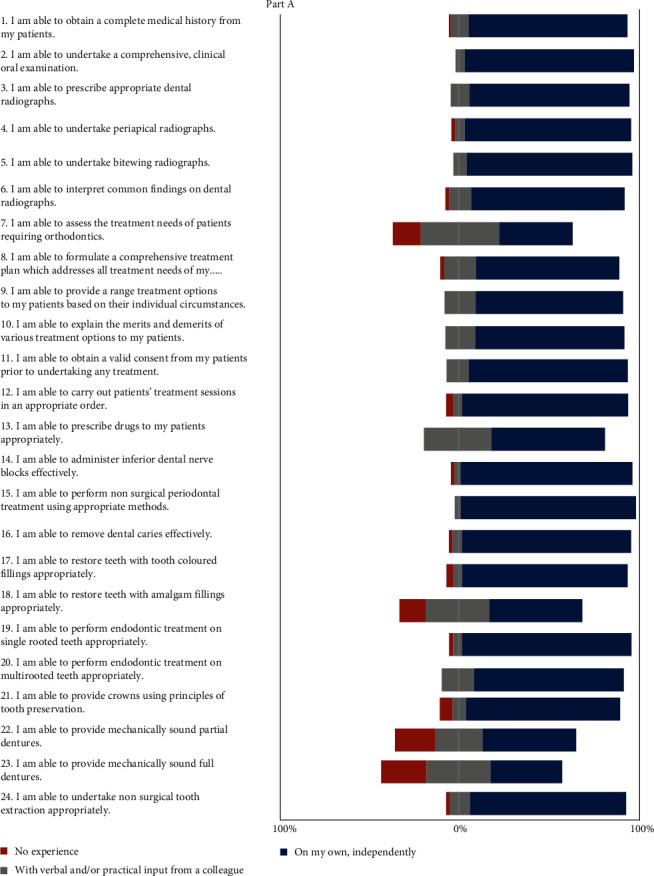
Responses to part A of questionnaire related to clinical skills.

Out of a batch of 64 final-year students, 58 students completed the questionnaire fully, comprising 46 females and 12 males (Figure 1). The data supporting the findings of this study are openly available upon reasonable request. After evaluating the responses to part A of the questionnaire (Figure 2), it was observed that most students (89.7%) could obtain a complete medical history, and nearly all (94.8%) were able to undertake a comprehensive clinical oral examination. About 89.7% of students could judge and choose the appropriate radiograph, and 93% could undertake periapical radiographs and take bitewing radiographs. Most students (about 86.2%) expressed confidence in interpreting radiographic findings independently, and 80.7% were able to plan a comprehensive treatment that met patients' needs. However, less than half (41.4%) could interpret the treatment needs of patients requiring orthodontic care.

Most students (82.5%) could provide an array of treatment options, and 84.2% could correctly explain the merits and demerits of each treatment option. Prior to carrying out any treatment, about 87.7% obtained the patient's consent, and 91.2% could undertake treatment sessions in a well-orchestrated order. More than half (62.5%) of the students could prescribe drugs independently, while 37.5% required verbal or practical input.

Almost all (94.7% and 96.5%) final-year students were confident in administering the inferior alveolar dental nerve block and performing nonsurgical periodontal treatment using appropriate methods, respectively. About 93% of students could remove dental caries lesions, and 91.2% could restore teeth with tooth-colored fillings, but only 50.9% could restore teeth with amalgam. Furthermore, 82.5% of students reported that they were able to perform endodontic treatment on multirooted teeth without supervision, while almost all (93%) could perform this procedure independently on single-rooted teeth. All students had experienced performing endodontic treatment on multirooted teeth, but 1.8% had no experience with single-rooted teeth.

A considerable number of students (85.7%) could provide crowns, but only half (51.9%) reported the ability to provide sound partial dentures independently, and only 39.3% could provide full dentures. About 25.9% and 35.7% needed guidance to provide sound partial and full dentures, respectively. A large number of students (87.5%) could undertake nonsurgical extractions.

**Figure 3:**
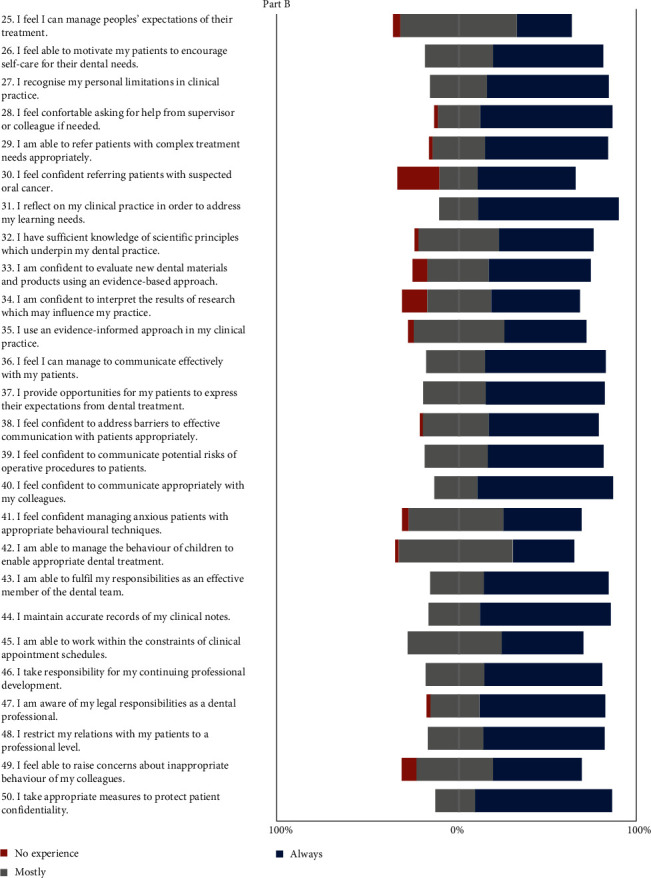
Responses to part B of questionnaire related to soft skills.

Evaluating part B of the filled questionnaire (Figure 3), it was found that only 31% of students could always manage patients' expectations of their treatment, and less than two-thirds (62.1%) could motivate patients to encourage self-care for their dental needs. About 68.4% of students could recognize and understand their personal limitations in clinical practice, and most (74.1%) felt comfortable asking for help from a supervisor or colleague.

Regarding the referral of patients with complex treatment needs and oral cancer patients, only 69% and 55.2% of students, respectively, expressed confidence. A considerable group of students (79.3%) could reflect on their clinical practice to address their learning needs. A little more than half (52.6% and 56.9%, respectively) expressed that they had sufficient knowledge that underpins dental practice and that they were confident in evaluating new dental materials and products using an evidence-based approach. Only half of the students were confident in interpreting the results of their research to influence their practice, and less than half (46.6%) used an evidence-informed approach in their clinical practice.

Around two-thirds (67.2% and 66.7%) of students conveyed that they could effectively communicate with patients and provide opportunities for patients to express their expectations from dental treatment, respectively. The responses related to communication revealed that 61.4% of students felt confident in addressing barriers to effective communication with patients. More than half (64.9%) felt confident in communicating the potential risks of operative procedures to patients, and about 75.4% felt confident in communicating with colleagues.

Moreover, 43.9% of the students could always and 52.6% could mostly manage anxious patients using appropriate behavioral techniques. About 33.9% could always and 64.3% could mostly manage the behavior of children to enable appropriate treatment. A sizeable number of students (70.2%) were confident in fulfilling their responsibilities as effective members of the dental team, and 73.7% always maintained accurate records of their clinical notes. Less than half (46.6%) were always able to work within the constraints of clinical appointment schedules, but two-thirds (66.7%) took responsibility for continuing dental professional development. Most (70.7%) students were aware of their legal responsibilities, but 1.7% were not aware at all. About 69% of the study group maintained professional relationships with their patients, and no one crossed professional boundaries. Exactly half (50%) felt they could raise concerns about inappropriate behavior from colleagues, while 7.1% expressed no confidence in doing so. Almost all the graduating students took appropriate measures to protect patient confidentiality, and none of the students breached patient confidentiality.

## 6. Discussion

The current questionnaire-based study was conducted during the peak of the COVID-19 pandemic, a period that significantly impacted dental education globally. Traditional face-to-face clinical tasks, supervised patient interactions, and student training were replaced by online teaching due to lockdown measures. As the situation improved, teaching gradually transitioned to a hybrid mode combining online and onsite activities, initially focusing on artificial simulators and eventually reintroducing nonaerosolized patient procedures with stringent cross infection protocols. Throughout, safety measures such as physical distancing and mask-wearing were strictly observed among students on campus.

While a student may possess the manual dexterity to perform dental procedures, their perception of competence and self-belief in their abilities are equally crucial [11]. We sought to assess this self-perceived preparedness among final-year dental students at ODC during the unprecedented COVID-19 pandemic. Participants completed validated questionnaires (DU-PAS) to evaluate their preparedness and competence in practicing unsupervised dental science.

The responses from the current study cohort revealed a higher proportion of female BDS students (~68.5%), consistent with trends seen in other years at ODC and in medical sciences globally. This phenomenon, often termed the “feminization of medical science,” is observed in institutions across Oman and in developed countries like the UK, Canada, and Australia [12]. Similar findings were noted in a study evaluating undergraduate dental students in Malaysia, where female participation was also predominant [6]. The big divide and imbalance in gender participation in academic careers needs to be corrected by maintaining parity in numbers.

Assessing the preparedness and self-perceived confidence of BDS V students (2020–2021 batch) during the COVID-19 pandemic using DU-PAS, part A focused on clinical skills while part B covered soft skills, including cognitive and behavioral attributes.

Based on the mean scores of current study respondents, dental undergraduates in Oman reported a preparedness level of 74.6% despite the challenges posed by the pandemic. In comparison, dental students in Pakistan reported a lower mean preparedness score (61.10%) compared to their counterparts in Malaysia (79.5%), the United Kingdom (74%), and Saudi Arabia (79.08%) in recent studies conducted before the pandemic [1, 7, 13, 14]. Notably, a study from Saudi Arabia published in 2023, involving an all-male dental school, indicated higher levels of preparedness despite the pandemic challenges. These confidence levels are self-perceived by students, reflecting differences in expectations between new dentists and their clinical supervisors. Supervisors often rate trainees as less prepared than trainees rate themselves across various learning outcomes [15].

Approximately 80% of BDS V students could independently obtain medical histories, conduct comprehensive clinical oral examinations, and perform and interpret different types of radiographs. A study from the UK, conducted before COVID-19, similarly showed students were confident in preliminary patient assessments but less so in prescribing and interpreting dental radiographs [1]. Another preCOVID study from Pakistan reported students' capability in taking medical histories but showed less expertise in dental radiography procedures [3]. ODC students' confidence in these foundational procedures during the pandemic likely stemmed from previous clinical teaching experiences in nonpandemic years, where they were actively involved in these procedures under supervision. In contrast, some other studies have highlighted the reliance on radiography departments for such procedures [3]. The students in ODC themselves undertake the radiography procedures during training years under supervision. Besides developing technical knowledge of radiographic procedures amongst ODC students, most of them could also interpret the radiographs. The readily quick interpretation was seen because these ‘about to graduate students' were taught the basic principles of oral radiology in BDS II. Additionally, the mode of basic radiology teaching amid the pandemic featured a Microsoft Teams online interface, bolstered with recorded videos and live demonstrations related to basic concepts prior to the start of clinical procedures.

Furthermore, more than 80% of ODC students trained during the challenging pandemic period could independently formulate treatment plans. This contrasts with findings from a study in a developing country before COVID-19, where students were less confident in comprehensive treatment planning [3]. The current study aligns with a Saudi Arabian study reporting high confidence levels among graduating students in treatment planning in 2020 [16].

During the pandemic, ODC students showed confidence in providing customized treatment options and explaining treatment merits and demerits to patients. They also demonstrated knowledge of conducting treatment sessions in an orderly fashion and the importance of obtaining patient consent prior to treatment, consistent with findings from preCOVID studies [1].

A survey study done evaluating the dental schools in the USA reports that there is minimal evidence of how treatment planning is incorporated into academic and clinical teaching. The treatment plan for the patients is completed during the second patient visit, and a number of these schools have decentralized treatment planning [17]. During the COVID-19 scenario, the didactic teaching of the basic precepts of dental practice, which included designing the treatment plan, communicating with the patient regarding the ethos of dentistry, etc., was taught online. As the hybrid classes started, the students were primarily asked to record the case histories of the patients in the clinics and were supposed to prepare the treatment plan themselves under supervision and safety conditions.

Nevertheless, the adopted remote type of teaching cannot replace face-to-face teaching, as evaluated in a study. The students at ODC liked the online lectures reported in this study [18]. In order to increase the attention of the students during the lectures, they were advised to always switch on their cameras. The students liked the camera-assisted face-to-face interaction and did express that the study material provided to them was easily accessible. Moreover, the uploaded video recording in their system served as the ready reckoner at any time, and this fact was expressed by the students in their online methodology teaching feedback forms.

Slightly more than half of the students could prescribe the drugs without any supervision. Moving forward, there is a need to shift from nonclinical pharmacology to clinical pharmacology education to enhance retention and application among students, as demonstrated in a study from the USA [19].

The final-year students felt less prepared in interpreting research and applying it to their clinical practice, as well as in using an evidence-based approach to evaluate dental materials and products and in referring patients suspected of oral cancer. These findings are in accordance with studies conducted using the DU-PAS scale in Pakistan, the United Kingdom, and Malaysia, respectively [1, 7, 13].

Just 41.4% of students felt competent enough to evaluate the orthodontic patients' treatment needs. This reflects a global inconsistency in orthodontic curriculum, learning outcomes, teaching content, and competency assessment methods across dental schools [19]. In ODC, the teaching of the subject of orthodontics is initiated in the prefinal and final years of BDS, and therefore it is incumbent to revamp the curriculum in tandem with the other taught clinical subjects.

Overall, the current study showed that students rated themselves highly in many clinical skills and procedures, including administering nerve blocks, performing nonsurgical extractions, nonsurgical periodontal treatments, and endodontic procedures on single and multirooted teeth. They also expressed confidence in providing crowns using the principles of tooth preservation. However, fewer students felt confident in procedures involving amalgam fillings and complete or partial dentures. ODC does not emphasize clinical teaching of amalgam filling due to environmental health concerns related to mercury emissions, aligned with the Minamata Convention of 2013 [20]. Students who lacked confidence in prosthodontic treatments received remedial online hands-on sessions.

Studies across 16 UK dental schools and Malaysian institutions during the COVID-19 era also reported students' lack of preparedness in complex clinical domains of dental treatment [21, 22]. A study from Jordan highlighted students' partial acceptance of online clinical training methods during the pandemic. Many preferred indirect supervision after graduation for their clinical work. More than 60% of final-year BDS students wanted to have indirect supervision for their clinical work in practice after graduation, whereas about 33% of them were unsure of the confidence they had gained in specific dental clinical abilities [23].

The specialized COVID online teaching method (SCOTM) featured theoretical online classes followed by comprehensive online clinical sessions covering all specialties. Students engaged in live demonstrations of clinical skills using dummies and extracted teeth mounted on artificial simulators, along with theoretical learning. This approach was well-received by students, who appreciated the interactive online format and the accessibility of recorded materials for review [24]. Digitalization needs to be introduced actively in clinical dental skills, which serves as an adjunct to traditional face-to-face teaching [25]. Many of the studies found in the literature were conducted during preCOVID times [1, 3, 4, 7, 11]. There were also many studies conducted during the COVID-19 period [5, 6, 10, 13–16, 18, 22–25]. There is a study recently published which examines the self-perceived preparedness of Turkish students across 10 universities after the pandemic [26].

The current study aims to examine the effect of the pandemic on student readiness. The study also indicated areas where students felt less prepared, such as interpreting research for clinical practice, applying evidence-based approaches to evaluate dental materials, and referring patients suspected of oral cancer, aligning with findings from studies using the DU-PAS scale in Pakistan, the UK, and Malaysia [1, 7, 13].

In conclusion, the current study provides insights into the self-perceived preparedness and confidence of final-year dental students during the COVID-19 pandemic. It underscores the resilience and adaptability of students in maintaining clinical skills amid unprecedented challenges. Future dental education should integrate lessons learned from pandemic responses to enhance preparedness for future healthcare crises while maintaining high educational standards.

### 6.1. Limitations of the Study

This study has several limitations that should be considered when interpreting the findings. First, the confidence levels reported by students may not always align with their actual clinical expertise, despite the reliability of the DU-PAS tool used. Second, the sample size was relatively small, consisting solely of final-year students (BDS V) from a single institution, ODC in Oman. Therefore, the generalizability of the findings may be limited.

Another limitation is the reliance on retrospective self-reported data from students, which can introduce response bias and inaccuracies in self-assessment. Strict confidentiality measures were followed throughout the study by faculty members at the same institution, but potential researcher bias cannot be completely ruled out. Selection bias is also a concern, as the study included only those students who successfully completed their final year during the pandemic period. Additionally, recall bias may have influenced the results, as students were asked to retrospectively assess their preparedness in a disrupted educational environment.

These limitations highlight the need for caution when interpreting the study's findings and suggest areas for future research to explore these factors more comprehensively across broader student populations and educational settings.

### 6.2. Recommendations for the Study

Given the limited sample size of this study, further research with a larger sample size and multicenter studies is recommended to improve the generalizability of the findings. Additionally, it is recommended to incorporate objective measures of clinical competence, such as faculty evaluations, standardized patient assessments, or performance on licensure exams, to validate students' self-perceptions.

Furthermore, evaluating long-term outcomes, such as performance on licensure exams and early career experiences, would provide valuable insights into the effectiveness of dental education during disruptive periods like the pandemic. Investigating the effectiveness of specific curricular interventions or support mechanisms aimed at addressing the identified gaps in student preparedness is also recommended. These steps would help enhance the understanding of how dental education can adapt and improve in response to challenges such as the COVID-19 pandemic.

## 7. Conclusion

Based on the findings of this study during the COVID-19 pandemic, undergraduate students at this standalone institution in the Sultanate of Oman appear to be prepared for independent practice, displaying higher confidence levels in performing simple dental procedures compared to more complex ones. However, deficiencies were noted in areas common across similar studies. There is a pressing need to revise and align the teaching curriculum to better reflect competencies and desired outcomes.

## Figures and Tables

**Table 1:** Three-point scale for part A of the DU-PAS.

Item	Score
No experience	0
With verbal and/or practical input from a colleague	1
On my own, independently	2

**Table 2:** Three-point scale for part B of the DU-PAS.

Item	Score
No experience	0
Mostly	1
Always	2

## Data Availability

The quantitative data used to support the findings of this study are included within the article. The data that support the findings of this study are available from the corresponding author, Triveni Nalawade, upon reasonable request.
